# Characterisation and prognostic implications of the 12-lead electrocardiogram in children with RASopathy-associated hypertrophic cardiomyopathy

**DOI:** 10.1136/heartjnl-2025-326268

**Published:** 2025-10-07

**Authors:** Olga Boleti, Angela Sunjaya, Ella Field, Gabrielle Norrish, Jennifer Tollit, Elena Cervi, Juan Pablo Kaski

**Affiliations:** 1 Centre for Paediatric Inherited and Rare Cardiovascular Disease, Institute of Cardiovascular Science, University College London, London, UK; 2 Centre for Inherited Cardiovascular Diseases, Great Ormond Street Hospital For Children NHS Foundation Trust, London, UK

## Abstract

**Background:**

The 12-lead ECG is a simple, inexpensive clinical tool with a key role in the assessment of patients with hypertrophic cardiomyopathy (HCM). The aims of this single centre, retrospective cohort study were to characterise ECG findings and to identify potential ECG predictors of major adverse cardiovascular events (MACE—cardiovascular mortality, resuscitated cardiac arrest, ventricular arrhythmias with haemodynamic compromise, appropriate implantable cardioverter defibrillator therapy or heart failure hospitalisation) in children with RASopathy-associated HCM (RAS-HCM).

**Methods:**

The resting 12-lead ECGs of 84 children with RAS-HCM were compared with those from 113 patients with sarcomeric HCM (s-HCM).

**Results:**

A significant proportion of ECGs in RAS-HCM had superior axis deviation (29.8% vs 2.5%, p value<0.001) and voltage criteria for right ventricular hypertrophy (52.4% vs 28.3%, p value<0.001), and a significantly lower prevalence of pathological Q waves (27.4% vs 47.8%, p value<0.001). Over a median follow-up period of 6.8 years (3.1–9.7), 19 patients (22.6%) with RAS-HCM suffered an MACE. Right atrial enlargement and ST segment changes>2 mm correlated with MACE on univariate analysis, with the latter remaining significant after adjustment in a multivariate model (adjusted relative risk (RR) 2.33, 95% CI 1.12 to 4.86, p value 0.024).

**Conclusion:**

These findings suggest that the 12-lead ECG may be a useful screening tool to distinguish RAS-HCM from s-HCM in everyday practice and could have potential implications for prediction of adverse outcomes.

## Introduction

 Hypertrophic cardiomyopathy (HCM) caused by disease-causing variants in genes regulating the RAS/MAPK pathway (RASopathy-associated HCM, RAS-HCM) accounts for up to 18% of childhood HCM^1^ and over 40% of infants (<1 year of age) with the disease.^2^ RAS-HCM has an earlier onset and is associated with higher heart failure hospitalisation rates,^3^ higher incidence of left ventricular outlet obstruction (LVOTO),^4^ significant diastolic dysfunction,^4^ cardiac interventions^5^ and overall worse survival than sarcomeric HCM (s-HCM),^5 6^ particularly in children younger than 2 years. Early diagnosis of RAS-HCM can result in prompt disease-specific management and may help to improve outcomes.^6 7^

The 12-lead ECG is a simple and non-invasive diagnostic tool, widely available even in low-resource settings, making it an effective screening tool for a wide range of cardiac conditions. In patients with HCM, ECG abnormalities can precede the development of left ventricular hypertrophy (LVH) by many years,^8^ and a normal ECG is usually only observed in fewer than 3% of paediatric HCM cases.^9^ Although typical ECG features in RAS-HCM are recognised in clinical practice,^7^ these have not been previously systematically evaluated and their role in predicting cardiovascular outcomes in this population is unknown. The aims of this study were to characterise the 12-lead ECG and to explore potential ECG predictors of adverse outcomes in children with RAS-HCM.

## Methods

### Study cohort

This was a single centre (Great Ormond Street Hospital, London, UK), retrospective cohort study. Patients<18 years old with a clinical and/or genetic diagnosis of a RASopathy syndrome (Noonan syndrome (NS), Noonan syndrome with multiple lentigines, Costello syndrome, cardiofaciocutaneous syndrome and Noonan-like syndrome with loose anagen hair (NS-LAH)) and HCM (see online supplemental file) were included from January 1990 to January 2024. Exclusion criteria were the absence of a baseline ECG within a year of the first date of assessment or a poor quality ECG precluding analysis (N=18). 12-lead ECGs from a separate cohort of patients (<18 years old) with a diagnosis of HCM secondary to a pathogenic or likely pathogenic s-HCM were used as a comparison group.

### Data collection

Clinical and genetic data were collected, including: demographics; genotype, baseline 12-lead ECG findings, corresponding echocardiographic parameters (within 6 months of ECG) and outcomes, including all-cause mortality, heart transplant, atrial/ventricular arrhythmias, cardiac arrest, LV septal myectomy, congestive heart failure (CHF) admission to hospital, implantable cardioverter defibrillator (ICD) implantation and appropriate therapies. A composite outcome of major adverse cardiac event (MACE) was created for analysis purposes, comprising: cardiovascular death, resuscitated cardiac arrest, ventricular arrhythmia with haemodynamic compromise, appropriate ICD therapy and CHF hospitalisation.

### ECG analysis

Systematic ECG analysis was carried out by two investigators (OB and AS). Details on ECG, echocardiographic and statistical analyses are reported in the online supplemental files.

## Results

### Patient demographics

84 patients with RAS-HCM were included and compared with 113 patients with s-HCM. The most common RASopathy diagnosis was NS (N=59, 70.2%). Pathogenic/likely pathogenic variants were most commonly found in *PTPN11* (N=25, 29.8%), followed by *RAF1* (11, 13.1%). In patients with s-HCM, the two most commonly implicated genes were *MYH7* in 53 (46.9%) and *MYBPC3* in 40 (35.4%) patients.

Patients with RAS-HCM had an overall younger median age at baseline assessment (1.0 years (0–3.5) vs 9.0 (3–13), p<0.001), and more commonly had concomitant congenital heart disease and valvular abnormalities (N=43 (51.2%) vs N=16 (14.2%), p<0.001); of whom 30 (35.7%) had pulmonary valve stenosis (PVS) as a sole defect or in combination with other defects (polyvalvulopathy N=35 (41.7%), atrial septal defect N=11 (13.1%), patent ductus arteriosus N=6 (7.1%)). The two groups had comparable maximal left ventricular wall thickness z-scores on echocardiogram, but patients with RAS-HCM had a larger left atrial diameter (LAd) (LAd z score 17.4 (9.4) vs +2.8 (2.8), p<0.001) and a higher proportion had concomitant right ventricular hypertrophy (RVH) (N=31 (50.0%) vs N=20 (26.9%), p<0.001). A detailed comparison of the baseline demographics, clinical and echocardiographic characteristics of the two groups is shown in table 1.

**Table 1:** Baseline demographics, clinical and echocardiographic characteristics

	Total	s-HCM	RAS-HCM	P value
	N=197	N=113	N=84	
Male	128 (65.0%)	73 (64.6%)	55 (65.5%)	0.90
FHx of HCM	56 (28.4%)	50 (44.2%)	6 (7.1%)	**<0.001**
Concomitant CHD	59 (29.9%)	16 (14.2%)	43 (51.2%)	**<0.001**
Age (years)	5.0 (1.0–11.0)	9.0 (3.0–13.0)	1.0 (0.0–3.5)	**<0.001**
Medication	91 (46.7%)	45 (39.8%)	46 (56.1%)	**0.025**
MLVWT (mm)	12.0 (8.0–18.0)	14.0 (9.0–20.3)	10.0 (7.0–15.0)	**<0.001**
MLVWT z score	10.7 (8.4)	10.3 (7.2)	11.6 (10.8)	0.43
LAD (mm)	25.5 (18.2–30.0)	24.2 (15.8–30.0)	27.0 (19.6–32.7)	0.36
LAD z score	5.9 (8.1)	2.8 (3.8)	17.4 (9.4)	**<0.001**
LVOTO	59 (36.2%)	19 (17.6%)	40 (72.7%)	**<0.001**
RVH	51 (65.0%)	20 (26.9%)	31 (50.0%)	**<0.001**

Statistically significant values are indicated in bold.

CHD, congenital heart defects; FHx, family history; HCM, hypertrophic cardiomyopathy; LAD, left atrial diameter; LVOTO, left ventricular outflow tract obstruction; MLVWT, maximal left ventricular wall thickness; RAS-HCM, RASopathy-associated HCM; RVH, right ventricular hypertrophy; s-HCM, sarcomeric HCM.

### ECG features in RAS-HCM

At baseline, the ECG of patients with RAS-HCM demonstrated a significantly higher prevalence of axis deviation (N=79 (65.5%) vs N=29 (35.4%), p value<0.001) compared with s-HCM, specifically superior axis deviation (N=25 (29.8%) vs N=3 (2.5%), p value<0.001). ECG evidence of atrial enlargement was more common in RAS-HCM (N=28 (33.3%) vs N=27 (23.8%), p value=0.006), as were voltage criteria for RVH (N=44 (52.4%) vs N=32 (28.3%), p value<0.001), with 28 out of 30 patients (93.3%) with PVS fulfilling voltage criteria for RVH. Voltage criteria for RVH on ECG did not correlate with echocardiographic presence of RVH (p=0.596), but showed an association with the presence of concomitant PVS (p<0.001). Patients with RAS-HCM had a significantly lower prevalence of pathological Q waves than patients with s-HCM (N=23 (27.4%) vs N=54 (47.8%), p value<0.001). ECG parameters were adjusted for potential confounders of age and concomitant CHD, but results remained significant (table 2). Intra and interobserver variability intraclass coefficient was>0.8 throughout.

**Table 2:** ECG characteristics in RAS-HCM vs s-HCM

	Total	s-HCM	RAS-HCM	P value	P value*	P value†
	N=197	N=113	N=84			
Abnormal axis	102 (51.8%)	73 (64.6%)	29 (34.5%)	**<0.001**	**0.002**	**0.042**
Type of axis deviation				**<0.001**	**0.008**	**0.006**
RAD	25 (12.7%)	15 (13.3%)	10 (11.9%)			
LAD	43 (21.8%)	24 (21.2%)	19 (22.6%)			
Superior axis	28 (14.2%)	3 (2.7%)	25 (29.8%)			
Evidence of atrial enlargement				**0.006**	**0.010**	**0.048**
RAE	26 (13.2%)	7 (6.2%)	19 (22.6%)			
LAE	20 (10.2%)	13 (11.5%)	7 (8.3%)			
Bi-AE	9 (4.6%)	7 (6.2%)	2 (2.4%)			
Pathological Q waves	77 (39.1%)	54 (47.8%)	23 (27.4%)	**<0.001**	**0.006**	**0.012**
Voltage criteria LVH	108 (54.8%)	68 (60.2%)	40 (47.6%)	0.080	0.132	0.320
Voltage criteria RVH	76 (38.6%)	32 (28.3%)	44 (52.4%)	**<0.001**	**0.002**	**0.003**
Conduction delay	84 (42.6%)	46 (40.7%)	38 (45.2%)	0.52	0.823	0.404
RBBB	10 (5.1%)	8 (7.1%)	2 (2.4%)			
LBBB	8 (4.1%)	4 (3.5%)	4 (4.8%)			
ST changes>2 mm	62 (31.5%)	41 (36.3%)	21 (25.0%)	0.092	0.087	0.250
TWI	85 (43.1%)	55 (48.7%)	30 (35.7%)	0.069	0.052	0.216
Giant T waves (>10 mm)	43 (21.8%)	26 (23.0%)	17 (20.2%)	0.64	0.401	0.652
Mean QTc (ms)	441.0 (32.9)	449.0 (31.3)	439.0 (35.3)	0.072	0.126	0.324
QT prolongation	29 (23.8%)	14 (23.3%)	25 (24.0%)	0.919	0.872	0.782
U waves	26 (13.2%)	12 (10.6%)	14 (16.7%)	0.21	0.087	0.654

Statistically significant values are indicated in bold.

* Adjusted for age.

† Adjusted for presence of concomitant congenital heart defect.

Bi-AE, bi-atrial enlargement; HCM, hypertrophic cardiomyopathy; LAD, left axis deviation; LAE, left atrial enlargement; LBBB, left bundle branch block; LVH, left ventricular hypertrophy; RAD, right axis deviation; RAE, right atrial enlargement; RAS-HCM, RASopathy-associated HCM; RBBB, right bundle branch block; RVH, right ventricular hypertrophy; s-HCM, sarcomeric HCM; TWI, T wave inversion.

### Correlation of ECG with MACE in RAS-HCM

**Figure 1:**
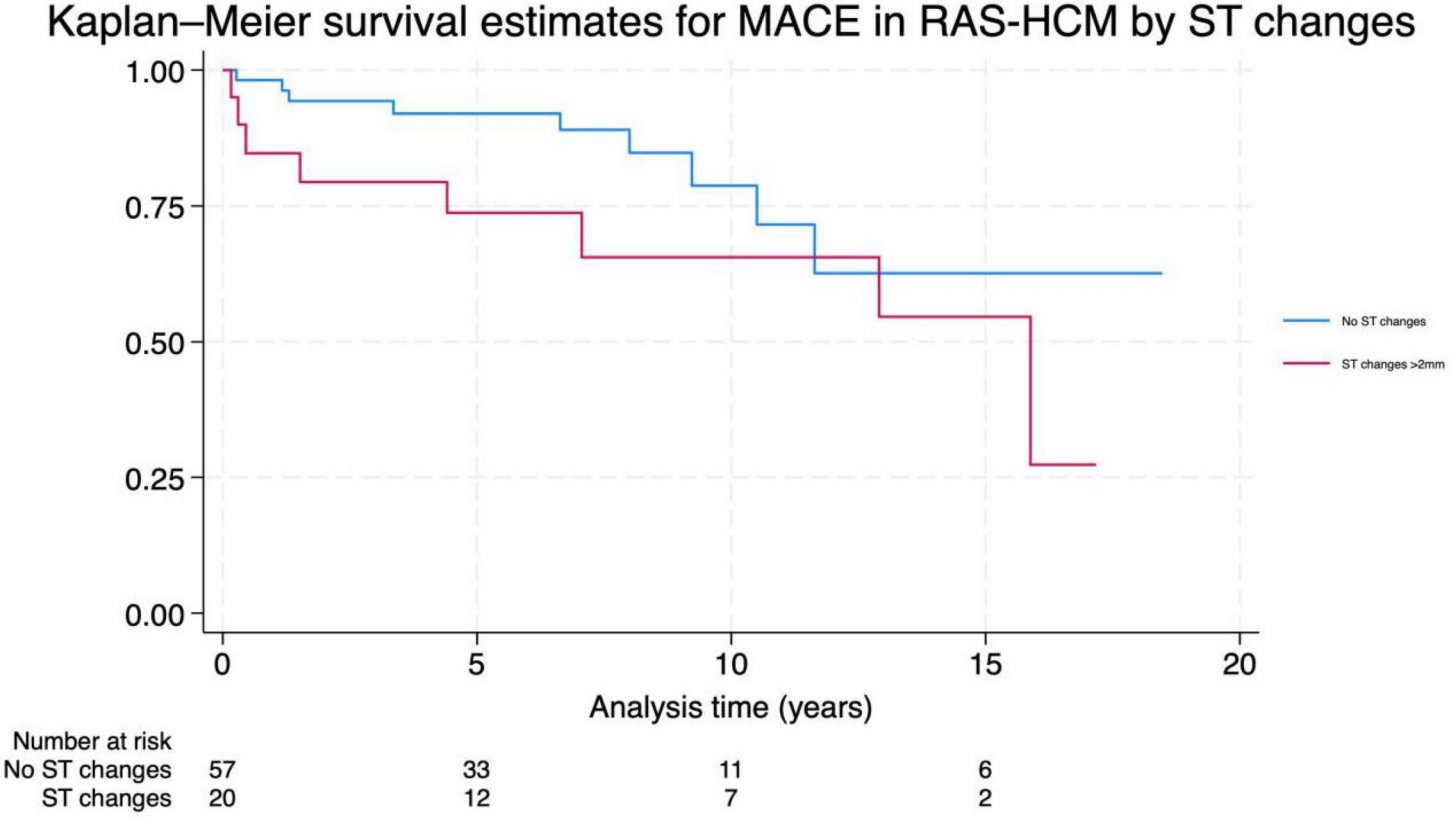
Kaplan-Meier survival curve for major arrhythmic cardiac events (MACE) in RASopathy-associated hypertrophic cardiomyopathy (RAS-HCM), grouped by the presence of ST segment changes>2 mm on ECG. Follow-up time in years.

Over a median follow-up period of 6.8 years (3.1–9.7), a total of 17 patients (20.2%) died of any cause in the RAS-HCM group, of whom 5 (5.9%) died of cardiac causes (2 CHF-related deaths, 2 sudden cardiac deaths (SCDs), 1 other cardiovascular-related death). There were a total of 19 (22.6%) MACE (7 CHF admissions, 5 cardiac-related deaths, 3 aborted cardiac arrests, 3 sustained ventricular tachycardia, 1 appropriate ICD therapy). On univariate analysis, right atrial enlargement and ST segment changes>2 mm correlated significantly with MACE. After adjustment in a multivariate model, only ST segment changes>2 mm remained significant (adjusted relative risk 2.33, 95% CI 1.12 to 4.86, p value 0.024) (table 3, figure 1).

**Table 3:** Logistic regression for ECG characteristics in RAS-HCM (N=84) and MACE (N=19)

	RR	95% CI		P value	Adjusted RR	95% CI		P value
Axis deviation	0.51	0.18	1.38	0.185				
RAD	2.50	0.92	6.79	0.072				
LAD	1.05	0.33	3.54	0.932				
Superior	0.84	0.34	2.09	0.712				
RAE	**2.95**	**1.28**	**6.76**	**0.011**	2.45	0.84	7.09	0.212
LAE	2.00	0.98	2.01	0.084				
Bi-AE	1.00	–	–	–				
Q waves present	2.16	0.33	14.32	0.425				
Voltage criteria LVH	1.51	0.68	3.38	0.313				
Voltage criteria RVH	1.56	0.68	3.57	0.294				
Conduction delay	1.35	0.62	2.97	0.463				
RBBB	2.28	0.54	9.69	0.264				
LBBB	1.11	0.19	6.36	0.906				
ST changes>2 mm	**2.70**	**1.27**	**5.73**	**0.010**	**2.33**	**1.12**	**4.86**	**0.024**
TWI	0.64	0.26	1.61	0.345				
Giant T waves (>10 mm)	1.82	0.81	4.08	0.147				
QTc (ms)	1.02	0.95	1.07	0.623				
QTc prolongation	4.21	0.64	7.82	0.124				
U waves	0.94	0.31	2.79	0.908				

Statistically significant values are indicated in bold.

Bi-AE, bi-atrial enlargement; LAD, left axis deviation; LAE, left atrial enlargement; LBBB, left bundle branch block; LVH, left ventricular hypertrophy; MACE, major adverse cardiac events; RAD, right axis deviation; RAE, right atrial enlargement; RAS-HCM, RASopathy-associated hypertrophic cardiomyopathy; RBBB, right bundle branch block; RR, relative risk; RVH, right ventricular hypertrophy; TWI, T wave inversion.

## Discussion

This study shows that children with RAS-HCM have distinct ECG features, including superior axis deviation, evidence of atrial enlargement and voltage criteria for RVH. ST segment changes on baseline ECG emerged as an independent predictor for MACE.

Superior axis deviation has long been considered to be a feature specific to RAS-HCM and is included as a ‘red flag’ raising suspicion of RASopathies as the underlying aetiology for paediatric HCM.^7^ However, this is, to our knowledge, the first study that describes the prevalence of this finding in comparison to children with s-HCM. Another notable difference is the high prevalence of RVH voltage criteria, which is in keeping with data from a recent study.^10^ RAS-HCM is known to have higher rates of biventricular involvement,^4 5^ which could be explained by the higher prevalence of concomitant congenital heart defects in RAS-HCM, specifically right heart lesions such as pulmonary valve stenosis; future studies to assess causality between electrocardiographic RVH and PVS in RAS-HCM are required.

There was a significant association between the presence of ST segment changes>2 mm with MACE in RAS-HCM. This is in contrast with ECG features in children with s-HCM, where no associations with MACE have been found,^9^ highlighting the differences between the two cohorts. Microvascular ischaemia has been associated with MACE in adults with HCM,^11^ but data in paediatric HCM populations, and in particular RAS-HCM, are lacking. The mechanisms of coronary ischaemia thought to play a part in s-HCM are also present in RAS-HCM, including microstructural abnormalities such as impaired coronary blood flow due to small vessel disease and microvascular dysfunction,^12^ haemodynamic alterations related to LVOTO,^6 7^ impaired diastolic function,^7^ myocardial hypercontractility^13^ and increased oxygen demand creating an energy mismatch.^14^ In addition, patients with RASopathy syndromes commonly have concomitant congenital heart defects,^6 7^ which may place an additional ischaemic burden on the myocardium, and additional contributing mechanisms such as coronary artery ectasia^15^ may also play a role. The assessment of microvascular disease in childhood HCM is challenging, due to the patchy nature of the disease and technical difficulties related to heart rate, but the finding that ST changes on the 12-lead ECG are associated with adverse outcomes suggests that further efforts to evaluate this are warranted.

### Limitations

This study is limited by the relatively small sample size which means that it may not be powered to detect potentially important differences in the ECG, specifically in exploring genotype–phenotype correlations. Given the retrospective study design, some clinical data may be incomplete, particularly in relation to genetic testing, which varies according to era, resulting in some patients with clinical features of a RASopathy syndrome not having a genetic diagnosis, despite review from a clinical geneticist. ST depression and elevation may represent different pathologies, so conclusions must be interpreted with caution. There may be a correlation of ST depression with myocardial strain which was not analysed in the present study. Due to a low event rate for adverse outcomes, MACE is a composite outcome encompassing cardiac mortality, heart failure and SCD equivalent events and we did not have the power to investigate risk factors for individual outcomes in isolation.

## Conclusion

This study demonstrates distinct ECG features in children with RAS-HCM, including superior axis deviation and voltage criteria for RVH. This could help distinguish RAS-HCM from s-HCM in clinical practice. ST segment changes are an independent predictor of MACE in this population, which could have potential implications for the prediction of adverse outcomes, but larger studies are needed to investigate this further.

## Supplementary material

10.1136/heartjnl-2025-326268 online supplemental file 1

## Data Availability

Data are available upon reasonable request.
