# Supplementary material for: Characterisation and prognostic implications of the 12-lead electrocardiogram in children with RASopathy-associated hypertrophic cardiomyopathy
Source: Heart. 2025 Oct 7;112(12):e326268. doi: 10.1136/heartjnl-2025-326268 (PMC13217015; doi:10.1136/heartjnl-2025-326268)
Supplement: online supplemental file 1 [file heartjnl-112-12-s001.docx]

**SUPPLEMENTAL METHODS**

**ECG analysis**

ECGs were analysed using normal paediatric reference values for age^1,2^. ECG criteria for left ventricular hypertrophy (LVH) and right ventricular hypertrophy (RVH) were based on the Sokolow-Lyon criteria^3^. The following findings were considered abnormal: axis deviation [left or right, defined according to reference values for age, with a QRS axis >180^o^ (extreme North-West axis) termed ‘superior QRS axis^4^’]; right atrial (RA) or left atrial (LA) dilatation; RVH and/or LVH^3^; left bundle branch block (LBBB)/right bundle branch block (RBBB); T wave inversion (TWI); corrected QT interval (QTc), according to the Bazzett formula^5^; and ST segment changes [depression (≥2mm in any lead) and elevation (≥2mm in leads V1-V3, or ≥1mm in all other leads]^6^. After the initial ECG review, 10% of ECGs were subjected to blinded analysis by the original investigator while a separate 10% underwent blinded analysis repeated by the other investigator, to assess intraobserver and interobserver variability, respectively.

**Echocardiographic analysis**

A diagnosis of hypertrophic cardiomyopathy (HCM) was defined as a left ventricular (LV) wall thickness ≥2 standard deviations above the body surface area‐corrected population mean (z score ≥2) that could not be explained solely by abnormal loading conditions^7^. Echocardiography was performed in line with the American Society of Echocardiography guidelines^8^. Maximal left ventricular wall thickness (MLVWT) was defined as the maximal myocardial thickness as measured by echocardiography in any of the LV segments^7^. Left ventricular outflow tract (LVOT) obstruction (LVOTO) was defined as a peak instantaneous gradient ≥ 30 mmHg^9^. Any degree of pulmonary valve stenosis has been evaluated in line with the European Society of Cardiology guidelines^10^. Cardiac dimensions were corrected for body surface area^11^ using previously published normative data^12^.

**Ethics**

This study complies with the Declaration of Helsinki. IRAS and local ethical approval was obtained with a waiver of informed consent for retrospective, anonymized data (IRAS project ID 182354, local R&D 15HC40).

**Statistical analysis**

The normality of data was tested using the Shapiro-Wilk method before selecting the appropriate statistical testing. Chi-square or Fischer exact tests were used for comparison of categorical variables and the Mann-Whitney U test for continuous variables. One way analysis of variance (ANOVA) was used to test for differences between ECG parameters and the most common RASopathy syndromes and genes (N≥5). Association between RAS-HCM ECG parameters and MACE was tested using binomial regression. Variables with a significant p-value were then inputted in a multivariate analysis and an adjusted relative risk (RR), 95% confidence interval (CI) and p-values calculated. Association between RAS-HCM ECG parameters and echocardiographic parameters was tested using binomial regression. Variants were adjusted for confounders using the Wald test. A significance level of 0.05 was used for all comparisons, while clinical importance was determined based on effect sizes and 95% CI. The intra and inter observer variability in the estimation of ECG features, were assessed using the intraclass coefficient (ICC), with values >.8 being considered as excellent. The coefficient of variation (defined as the ratio between the standard deviation of the mean of measurements and the mean between observers in percent) was also used as an additional assessment. Stata version 18 (StataCorp LLC, Lakeway Drive, College Station, Texas, US) was used for analysis and figures.

**SUPPLEMENTAL REFERENCES**

1. Rijnbeek PR, Witsenburg M, Schrama E, Hess J, Kors JA. New normal limits for the paediatric electrocardiogram. *Eur Heart J*. Apr 2001;22(8):702-11. doi:10.1053/euhj.2000.2399

2. Dickinson DF. The normal ECG in childhood and adolescence. *Heart*. Dec 2005;91(12):1626-30. doi:10.1136/hrt.2004.057307

3. Sokolow M, Lyon TP. The ventricular complex in left ventricular hypertrophy as obtained by unipolar precordial and limb leads. *Am Heart J*. Feb 1949;37(2):161-86. doi:10.1016/0002-8703(49)90562-1

4. Shaw NJ, Godman MJ, Hayes A, Sutherland GR. Superior QRS axis in ventricular septal defect. *Br Heart J*. Oct 1989;62(4):281-3. doi:10.1136/hrt.62.4.281

5. BAZETT HC. AN ANALYSIS OF THE TIME-RELATIONS OF ELECTROCARDIOGRAMS. *Annals of Noninvasive Electrocardiology*. 1997;2(2):177-194. doi:<https://doi.org/10.1111/j.1542-474X.1997.tb00325.x>

6. Rautaharju PM, Surawicz B, Gettes LS, et al. AHA/ACCF/HRS recommendations for the standardization and interpretation of the electrocardiogram: part IV: the ST segment, T and U waves, and the QT interval: a scientific statement from the American Heart Association Electrocardiography and Arrhythmias Committee, Council on Clinical Cardiology; the American College of Cardiology Foundation; and the Heart Rhythm Society. Endorsed by the International Society for Computerized Electrocardiology. *J Am Coll Cardiol*. Mar 17 2009;53(11):982-91. doi:10.1016/j.jacc.2008.12.014

7. Elliott PM, Anastasakis A, Borger MA, et al. 2014 ESC Guidelines on diagnosis and management of hypertrophic cardiomyopathy: the Task Force for the Diagnosis and Management of Hypertrophic Cardiomyopathy of the European Society of Cardiology (ESC). *Eur Heart J*. Oct 14 2014;35(39):2733-79. doi:10.1093/eurheartj/ehu284

8. Mitchell C, Rahko PS, Blauwet LA, et al. Guidelines for Performing a Comprehensive Transthoracic Echocardiographic Examination in Adults: Recommendations from the American Society of Echocardiography. *J Am Soc Echocardiogr*. Jan 2019;32(1):1-64. doi:10.1016/j.echo.2018.06.004

9. Kaski JP, Norrish G, Gimeno Blanes JR, et al. Cardiomyopathies in children and adolescents: aetiology, management, and outcomes in the European Society of Cardiology EURObservational Research Programme Cardiomyopathy and Myocarditis Registry. *Eur Heart J*. Apr 21 2024;45(16):1443-1454. doi:10.1093/eurheartj/ehae109

10. Baumgartner H, Hung J, Bermejo J, et al. Echocardiographic assessment of valve stenosis: EAE/ASE recommendations for clinical practice. *Eur J Echocardiogr*. Jan 2009;10(1):1-25. doi:10.1093/ejechocard/jen303

11. Haycock GB, Schwartz GJ, Wisotsky DH. Geometric method for measuring body surface area: a height-weight formula validated in infants, children, and adults. *J Pediatr*. Jul 1978;93(1):62-6. doi:10.1016/s0022-3476(78)80601-5

12. Lopez L, Colan S, Stylianou M, et al. Relationship of Echocardiographic Z Scores Adjusted for Body Surface Area to Age, Sex, Race, and Ethnicity: The Pediatric Heart Network Normal Echocardiogram Database. *Circ Cardiovasc Imaging*. Nov 2017;10(11)doi:10.1161/circimaging.117.006979
